# Antimicrobial-drug use and changes in resistance in Streptococcus pneumoniae.

**DOI:** 10.3201/eid0605.000519

**Published:** 2000

**Authors:** D J Diekema, A B Brueggemann, G V Doern

**Affiliations:** University of Iowa College of Medicine, Iowa City, Iowa 52242, USA. daniel-diekema@uiowa.edu

## Abstract

Resistance of Streptococcus pneumoniae to antimicrobial drugs is increasing. To investigate the relationship between antimicrobial use and susceptibility of S. pneumoniae isolates at 24 U.S. medical centers, we obtained data on outpatient antimicrobial-drug use for the regions surrounding 23 of these centers. We found an association between decreased penicillin susceptibility and use of beta-lactam antimicrobial drugs.


### Antimicrobial-Drug Use and Changes in Resistance in *Streptococcus pneumoniae*

**Daniel J. Diekema, Angela B. Brueggemann, Gary V. Doern**
University of Iowa College of Medicine, Iowa City, Iowa

Resistance of *Streptococcus pneumoniae* to antimicrobial drugs is increasing. To investigate the relationship between antimicrobial use and susceptibility of *S. pneumoniae* isolates at 24 U.S. medical centers, we obtained data on outpatient antimicrobial-drug use for the regions surrounding 23 of these centers. We found an association between decreased penicillin susceptibility and use of beta-lactam antimicrobial drugs.

Resistance of *Streptococcus pneumoniae* to penicillin and other beta-lactams is increasing worldwide (1-4). The major mechanism of resistance involves the introduction of mutations in genes encoding penicillin-binding proteins (5). Selective pressure is thought to play an important role, and use of beta-lactam antibiotics has been implicated as a risk factor for infection and colonization (6-14). Wide geographic spread of resistant clones has been described (3,15). However, the effect of geographic patterns of antimicrobial-drug use on the emergence and spread of resistance is not known.

We performed two previous surveillance studies of *S. pneumoniae* isolated at medical centers in the United States, one in 1994-95 (16), the other in 1997-98 (17). We report the relationship between antimicrobial-drug use in the geographic areas surrounding these medical centers and the change in penicillin resistance of *S. pneumoniae* over a 3-year period.

### The Study

Multicenter national surveillance of *S. pneumoniae* was performed from November 1994 to April 1995 (16) and again from November 1997 to April 1998 (17). All isolates during these two surveillance studies were recovered from consecutive nonhospitalized patients from either the lower respiratory tract or a sterile site (blood or cerebrospinal fluid). Briefly, isolates were transported from study centers to a central laboratory, where they were confirmed as *S. pneumoniae* by conventional identification methods (16). Susceptibility testing was performed by the reference broth microdilution method recommended by the National Committee for Clinical Laboratory Standards (NCCLS) (18). Susceptibility was determined by using the established NCCLS breakpoints (19). For penicillin, breakpoints of 0.1 to 1.0 µg/mL for intermediate and $\geq$2 µg/mL for resistant were used; for this analysis, both intermediate and resistant categories were considered resistant.

Twenty-four medical centers were surveyed during both study periods. For 23 of these centers, data for outpatient antimicrobial-drug use were obtained for the surrounding metropolitan statistical area. These data were expressed in terms of number of prescriptions written per 100,000 population per month during the 48-month period that included the two surveillance studies (20). This period (May 1994 through April 1998) included four consecutive respiratory virus seasons. We divided the 23 medical centers into high-, intermediate-, and low-use centers for each antimicrobial-drug class. With the change in penicillin resistance as the dependent variable of interest, we used one-way ANOVA to compare mean change in resistance to penicillin between high-, intermediate-, and low-use centers. We then analyzed covariance models to evaluate the relationship between antimicrobial-drug use categories and changes in penicillin resistance. Alpha was set at 0.05, and all p-values were two-tailed.

We compiled the penicillin and erythromycin susceptibility test results for *S. pneumoniae*

Address for correspondence: Daniel J. Diekema, Medical Microbiology Division, C606 GH, Department of Pathology, University of Iowa College of Medicine, Iowa City, Iowa 52242; fax: 319-356-4916; e-mail: daniel-diekema@uiowa.edu.

isolates collected in 1994-95 and 1997-98 from all 23 centers (Table 1). Overall, penicillin nonsusceptibility (MIC $\geq$ 0.1 µg/mL) increased by 8.9%. In 1994-95, 269 (22.2%) of the 1,211 *S. pneumoniae* isolates were intermediate or fully resistant to penicillin, while in 1997-98, 337 (31.1%) of the 1,083 isolates were in these categories. When the change in percent penicillin susceptibility at each

Table 1. Change in resistance[a] among *Streptococcus pneumoniae* isolates at 23 U.S. medical centers, 1994-95 and 1997-98

| Medical center | No. of isolates | Study period | Erythromycin | Change (%) | Penicillin I + R[b] | Change (%) |
|---|---|---|---|---|---|---|
| Seattle, WA | 37 | 1994-95 | 5.4 | | 35.1 | |
| | 50 | 1997-98 | 30.0 | 24.6 | 38.0 | 2.9 |
| Denver, CO | 62 | 1994-95 | 3.2 | | 14.5 | |
| | 26 | 1997-98 | 7.7 | 4.5 | 15.4 | 0.9 |
| Phoenix, AZ | 57 | 1994-95 | 12.3 | | 40.4 | |
| | 54 | 1997-98 | 35.2 | 22.9 | 40.7 | 0.3 |
| Houston, TX | 63 | 1994-95 | 22.2 | | 25.4 | |
| | 48 | 1997-98 | 43.8 | 21.6 | 64.6 | 39.2 |
| Dallas, TX | 58 | 1994-95 | 6.9 | | 22.4 | |
| | 36 | 1997-98 | 27.8 | 20.9 | 30.5 | 8.1 |
| Rochester, MN | 35 | 1994-95 | 8.6 | | 14.2 | |
| | 48 | 1997-98 | 20.8 | 12.2 | 22.9 | 8.7 |
| Milwaukee, WI | 65 | 1994-95 | 18.5 | | 33.8 | |
| | 55 | 1997-98 | 10.9 | -7.6 | 20.0 | -13.8 |
| Evanston, IL | 49 | 1994-95 | 8.2 | | 14.3 | |
| | 35 | 1997-98 | 14.3 | 6.1 | 14.3 | 0.0 |
| Chicago, IL | 41 | 1994-95 | 17.1 | | 34.1 | |
| | 41 | 1997-98 | 19.5 | 2.4 | 19.5 | -14.6 |
| Indianapolis, IN | 63 | 1994-95 | 7.9 | | 20.7 | |
| | 55 | 1997-98 | 18.2 | 10.3 | 25.5 | 4.8 |
| St. Louis, MO | 57 | 1994-95 | 8.9 | | 24.6 | |
| | 55 | 1997-98 | 12.7 | 3.8 | 29.1 | 4.5 |
| Detroit, MI | 63 | 1994-95 | 6.3 | | 19.0 | |
| | 60 | 1997-98 | 10.0 | 3.7 | 30.0 | 11.9 |
| Cleveland, OH | 42 | 1994-95 | 11.9 | | 19.0 | |
| | 60 | 1997-98 | 20.0 | 8.1 | 23.2 | 4.2 |
| Philadelphia, PA | 47 | 1994-95 | 2.1 | | 2.1 | |
| | 42 | 1997-98 | 11.9 | 9.8 | 21.4 | 19.3 |
| Syracuse, NY | 23 | 1994-95 | 8.7 | | 8.7 | |
| | 50 | 1997-98 | 8.0 | -0.7 | 20.0 | 11.3 |
| Rochester, NY | 58 | 1994-95 | 6.9 | | 10.4 | |
| | 50 | 1997-98 | 12.0 | 5.1 | 20.0 | 9.6 |
| New York, NY | 64 | 1994-95 | 4.7 | | 12.6 | |
| | 53 | 1997-98 | 3.8 | -0.9 | 20.8 | 8.2 |
| Hartford, CT | 61 | 1994-95 | 3.3 | | 8.2 | |
| | 51 | 1997-98 | 7.8 | 4.5 | 27.4 | 19.2 |
| Washington, DC | 60 | 1994-95 | 13.3 | | 23.3 | |
| | 28 | 1997-98 | 28.6 | 15.3 | 35.7 | 12.4 |
| Chapel Hill, NC | 60 | 1994-95 | 10.0 | | 31.7 | |
| | 49 | 1997-98 | 38.8 | 28.8 | 57.1 | 25.4 |
| Decatur, GA | 61 | 1994-95 | 23.0 | | 36.1 | |
| | 52 | 1997-98 | 26.9 | 3.9 | 44.2 | 8.1 |
| Mobile, AL | 68 | 1994-95 | 16.2 | | 20.6 | |
| | 58 | 1997-98 | 37.9 | 21.7 | 41.3 | 20.7 |
| Miami, FL | 17 | 1994-95 | 5.9 | | 52.9 | |
| | 27 | 1997-98 | 29.6 | 23.7 | 51.8 | -1.1 |
| TOTAL | 1,211 | 1994-95 | 10.2 | | 22.2 | |
| | 1,083 | 1997-98 | 20.6 | 10.4 | 31.1 | 8.9 |

[a]Includes both intermediate- and high-level resistance to penicillin and erythromycin.
[b]I + R = both intermediate and fully resistant.

center was considered, the overall mean increase in penicillin resistance was 8.3% (-14.6% to 39.2%) among the 23 centers that participated in both surveys (Table 1).

Antimicrobial-drug use data for beta-lactams, tetracyclines, quinolones, and macrolides were calculated for the high-, intermediate-, and low-use tertiles in our analysis (Table 2). The mean increase in penicillin resistance was compared among high-, intermediate-, and low-use centers for the major antibiotic classes (Table 3). The beta-lactams were most strongly associated with an increase in penicillin resistance (2.8%, 8.8%, and 13.3% increases in low-, intermediate-, and high-use tertiles, respectively, p=0.20).

Univariate analysis of covariance was performed, with change in penicillin resistance as the dependent variable and the antimicrobial-drug use category for each antimicrobial-drug class as independent variables. When all classes for which data were available (beta-lactams, tetracyclines, macrolides, and quinolones) were entered into a model, only the macrolides and beta-lactams were statistically significant (p< 0.1) as explanatory variables and were therefore included in the final model (Table 4). Higher beta-lactam use was strongly associated with increased resistance to penicillin (F=8.7, p=0.008). Conversely, higher macrolide use was associated with decreased resistance to penicillin (F=5.4, p=0.031). The overall model explained a significant amount of the variance in penicillin resistance at these 23 centers (F=4.8, p=0.02).

Table 2. Prescriptions for antibiotics at medical centers with high, intermediate, and low antimicrobial-drug use[a]

| Class/tertile | Mean | Median | Range | SD |
|---|---|---|---|---|
| Beta-lactams | | | | |
| High | 1,640 | 1,620 | 1,186-2,557 | 411 |
| Intermediate | 1,027 | 1,040 | 948-1,136 | 69 |
| Low | 859 | 870 | 777-917 | 51 |
| Macrolides | | | | |
| High | 929 | 865 | 800-1,286 | 166 |
| Intermediate | 738 | 722 | 687-787 | 35 |
| Low | 609 | 623 | 528-673 | 52 |
| Quinolones | | | | |
| High | 282 | 258 | 222-424 | 63 |
| Intermediate | 197 | 200 | 177-216 | 16 |
| Low | 143 | 146 | 91-170 | 27 |
| Tetracyclines | | | | |
| High | 77 | 75 | 61-100 | 15 |
| Intermediate | 56 | 58 | 50-59 | 3 |
| Low | 33 | 34 | 25-45 | 7 |

[a]All values are expressed in units of mean number of prescriptions per 100,000 population per month during the period between the two surveillance studies (May 1994-April 1998).

Table 3. Mean increase in percent penicillin resistance[a] of *Streptococcus pneumoniae* by category[b] of antimicrobial-drug use

| Class | High | Inter-mediate | Low | p-value[c] |
|---|---|---|---|---|
| Beta-lactams | 13.3 | 8.8 | 2.8 | 0.20 |
| Quinolones | 13.0 | 6.3 | 5.3 | 0.39 |
| Macrolides | 4.0 | 12.4 | 8.9 | 0.39 |
| Tetracyclines | 5.3 | 7.7 | 11.8 | 0.56 |
| All classes | 13.3 | 3.3 | 7.6 | 0.27 |

[a]Includes both intermediate- (MIC 0.12-1 µg/mL) and high-level (MIC ≥2 µg/mL) resistance to penicillin.
[b]Each center was categorized by total number of outpatient prescriptions for the antimicrobial class per 100,000 population per month in the surrounding metropolitan statistical area.
[c]One-way ANOVA p-value, two-tailed.

Table 4. Analysis of covariance model, with change in penicillin resistance at each of the 23 medical centers as the dependent variable

| Source | Type III sums of squares | Parameter estimate (*B*) | F | p-value |
|---|---|---|---|---|
| Overall model | 990[a] | | 4.8 | 0.02 |
| Intercept | 56 | 4.5 | 0.5 | 0.47 |
| β-lactam use | 893 | 8.6 | 8.7 | 0.008 |
| Macrolide use | 553 | -6.7 | 5.4 | 0.031 |
| Error | 2,054 | | | |
| Total | 4,616 | | | |
| Corrected total | 3,045 | | | |

[a]$R^2$ = 0.325.

A separate analysis showed no significant association between beta-lactam, macrolide, quinolone, or tetracycline use and change in the percentage of erythromycin resistance. However, an overall increase in erythromycin resistance was observed (Table 1).

### Conclusions

Numerous studies have associated antimicrobial-drug use patterns in hospitals with the emergence of resistance among nosocomial pathogens (21-25). However, *S. pneumoniae* is usually acquired outside the hospital environment; therefore, establishing a relationship between antimicrobial-drug use and resistance requires outpatient data, as well as susceptibility test results. A large-scale study of this type is costly and difficult to perform in the United States, given the problems inherent in collecting accurate data from multiple outpatient settings. To generate hypotheses and support the planning of such a study, we used data collected for other purposes to explore the relationship between outpatient antimicrobial-drug use and resistance among *S. pneumoniae* isolates.

We found an association between the outpatient use of beta-lactam antimicrobial drugs in metropolitan areas and changes in the penicillin susceptibility of *S. pneumoniae* isolates sampled from tertiary care centers in those metropolitan areas. Determining whether this association is spurious or causal requires further investigation, given the limitations of our study design. Since information about each patient's previous antimicrobial-drug use was not available, we were unable to make a direct connection between patient use and risk for resistance. Furthermore, since antimicrobial-drug use data are presented as the total number of prescriptions per month in the population, the data may not accurately reflect use. Patient compliance, dosage prescribed, and duration of antibiotic use may differ from region to region. In addition, the data are for large populations, and the *S. pneumoniae* isolates represent a small sample from one study center in each metropolitan statistical area. These samples may not accurately reflect the true prevalence of resistance in the study population. For this reason, we grouped the study centers into tertiles on the basis of use, to decrease the impact of a small number of resistant isolates at a single study center. Finally, this analysis was retrospective. These surveillance surveys were not designed to evaluate the association between antimicrobial-drug use and changing resistance patterns among *S. pneumoniae*.

However, the use of antimicrobial agents in a population would be expected to contribute to the emergence and spread of resistance within that population, and our data support this hypothesis for beta-lactam use and penicillin resistance. The fact that beta-lactam use was associated with increased penicillin, but not erythromycin, resistance among pneumococcal isolates in our study suggests a specific association. Furthermore, this positive association with penicillin resistance was not seen for antimicrobial-drug classes other than the beta-lactams; neither was resistance associated with the total number of antimicrobial-drug prescriptions.

Erythromycin resistance also increased during our study. The lack of a strong association between use and erythromycin resistance may reflect the fact that beta-lactams were the most commonly prescribed in the metropolitan statistical areas we studied, and the impact of these drugs was therefore greater and easier to detect. In addition, a relationship between resistance to penicillin and resistance to virtually all other oral antimicrobial-drug classes has been described (2,16-17), making colinearity a potential problem in evaluating the impact of specific classes on resistance to a single antimicrobial agent or class. If the relationship between penicillin resistance and resistance to other antimicrobial-drug classes is due to the clonal spread of already multidrug-resistant strains (rather than emergence of resistance under antimicrobial pressure), the impact of a specific class of agent on the spread of a specific resistance in *S. pneumoniae* might vary by region, depending on the coresistance pattern of the predominant PRSP clones in that area.

Other investigators have reported an association between prescriptions for outpatients and rates of resistance in Western Europe (26), Hungary (27), and Iceland (28). In these studies, lower use of antimicrobial drugs in general and beta-lactams in particular is associated with lower rates of isolation of resistant strains. Our study supports this association and underscores the importance of implementing measures to decrease the inappropriate use of antibiotics in the outpatient setting (29).

Despite several limitations, our data support the hypothesis generated in previous studies that outpatient antimicrobial-drug use plays an important role in the development and spread of resistance. In future epidemiologic studies, antimicrobial-drug use should be carefully matched with resistance in well-defined populations and should include prospective evaluation of interventions to reduce the use of certain classes of antimicrobial agents for outpatients.

### Acknowledgments

The authors thank Holly K. Huynh, Paul R. Rhomberg, and Elizabeth M. Wingert for technical assistance.

This study was supported in part by an educational and research grant from Abbott Laboratories.

Dr. Diekema is clinical assistant professor in the Division of Infectious Diseases, Department of Internal Medicine, and the Division of Medical Microbiology, Department of Pathology, at the University of Iowa College of Medicine. He is associate hospital epidemiologist at University of Iowa Healthcare and hospital epidemiologist at the Iowa City Veterans Affairs Medical Center. His research interests focus on the epidemiology of antimicrobial-drug resistance among gram-positive bacterial pathogens.

### References

1. Butler JC, Hofmann J, Cetron MS, Elliott JA, Facklam RR, Breiman RF. The continued emergence of drug-resistant *Streptococcus pneumoniae* in the United States: an update from the Centers for Disease Control and Prevention's pneumococcal sentinel surveillance system. J Infect Dis 1996;174:986-93.
2. Doern GV, Pfaller MA, Kugler K, Freeman J, Jones RN. Prevalence of antimicrobial resistance among respiratory tract isolates of *Streptococcus pneumonae* in North America: 1997 results from the SENTRY antimicrobial surveillance program. Clin Infect Dis 1998;27:764-70.
3. Muñoz R, Coffey TJ, Daniels M, Dowson CG, Laible G, Casal J, et al. Intercontinental spread of a multiresistant clone of serotype 23F *Streptococcus pneumoniae*. J Infect Dis 1991;164:302-6.
4. Reichler MR, Rakovsky J, Sobotova A, Slacikova M, Hlavacova B, Hill B, et al. Multiple antimicrobial resistance of pneumococci in children with otitis media, bacteremia, and meningitis in Slovakia. J Infect Dis 1995;171:1491-6.
5. Laible G, Spratt BG, Hakenbeck R. Interspecies recombinational events during the evolution of altered PBP 2x genes in penicillin-resistant clinical isolates of *Streptococcus pneumoniae*. Mol Microbiol 1991;5:1993-2002.A
6. Arnold KE, Leggiadro RJ, Breiman RF, Lipman HB, Schwartz B, Appleton MA, et al. Risk factors for carriage of drug-resistant *Streptococcus pneumoniae* among Memphis, Tennessee, children. J Pediatr 1996;128:757-64.
7. Bedos JP, Chevret S, Chastang C, Geslin P, Regnier B, and the French Cooperative Pneumococcus Study Group. Epidemiologic features of and risk factors for infection by *Streptococcus pneumoniae* with diminished suceptibility to penicillin: findings of a French survey. Clin Infect Dis 1996;22:63-72.
8. Duchin JS, Breiman RF, Diamond A, Lipman HB, Block SL, Hedrick JA, et al. High prevalence of multidrug-resistant *Streptococcus pneumoniae* among children in a rural Kentucky community. Pediatr Infect Dis J 1995;14:745-50.
9. Ford KL, Mason EO, Kaplan SL, Lamberth L, Tillman J. Factors associated with middle ear isolates of *Streptococcus pneumoniae* resistant to penicillin in a children's hospital. J Pediatr 1991;119:941-4.
10. Nava JM, Bella F, Garau J, Lite J, Morera MA, Marti C, et al. Predictive factors for invasive disease due to penicillin-resistant *Streptococcus pneumoniae*: a population-based study. Clin Infect Dis 1994;19:884-90.
11. Pallares R, Gudiol F, Linares J, Ariza J, Rufi G, Margui L, et al. Risk factors and response to antibiotic therapy in adults with bacteremic pneumonia caused by penicillin-resistant pneumococci. N Engl J Med 1987;317:18-22.
12. Reichler MR, Allphin AA, Breiman RF, Schreiber JR, Arnold JE, McDougal LK, et al. The spread of multiply resistant *Streptococcus pneumoniae* at a day care center in Ohio. J Infect Dis 1992;166:1346-53.
13. Tan TQ, Mason EO, Kaplan SL. Penicillin-resistant systemic pneumococcal infections in children: a retrospective case-control study. Pediatrics 1993;92:761-7.
14. Soares S, Kristinsson KG, Musser JM, Tomasz A. Evidence for the introduction of a multiresistant clone of serotype 6B *Streptococcus pneumoniae* from Spain to Iceland in the late 1980's. J Infect Dis 1993;168:158-63.
15. Welby PL, Keller DS, Cromien JL, Tebas P, Storch G. Resistance to penicillin and non-beta-lactam antibiotics of *Streptococcus pneumoniae* at a children's hospital. Pediatr Infect Dis 1994;13:281-7.
16. Doern GV, Brueggemann A, Holley HP, Rauch AM. Antimicrobial resistance of *Streptococcus pneumoniae* recovered from outpatients in the United States during the winter months of 1994 to 1995: results of a 30-center national surveillance study. Antimicrob Agents Chemother 1996;40:1208-13.
17. Doern GV, Brueggemann AB, Huynh H, Wingert E, Rhomberg P. Antimicrobial resistance with *Streptococcus pneumoniae* in the United States, 1997-98. Emerg Infect Dis 1999;5:757-65.
18. Methods for dilution antimicrobial susceptibility tests for bacteria that grow aerobically. Approved standard M7-A4. Wayne (PA): National Committee for Clinical Laboratory Standards; 1997.
19. Performance standards for antimicrobial susceptibility testing. Supplemental tables, M100-S8. Wayne (PA): National Committee for Clinical Laboratory Standards; 1998.
20. BW Healthwire. IMS Health annual data show expanding pharmaceutical market growth. Biomed Pharmacother 1999; 53:290-2.
21. Gaynes R. The impact of antimicrobial use on the emergence of antimicrobial-resistant bacteria in hospitals. Infect Dis Clin North Am 1997;11:757-65.
22. Gerding DN, Larson TA. Resistance surveillance programs and the incidence of gram-negative bacillary resistance to amikacin from 1967-1985. Am J Med 1986;80:22-8.
23. McGowan JE. Antimicrobial resistance in hospital organisms and its relation to antibiotic use. Rev Infect Dis 1983;5:1033-48.
24. Monnet D, Gaynes R, Tenover F, McGowan JE, ICARE Pilot Hospitals. Ceftazidime-resistant *Pseudomonas aeruginosa* and ceftazidime usage in NNIS hospitals: preliminary results of Project ICARE, Phase one. Infect Control Hosp Epidemiol 1995;4(Suppl):19.
25. Muscato JJ, Wilbur DW, Stout JJ, Fahrlender RA. An evaluation of the susceptibility patterns of gram-negative organisms isolated in cancer centers with aminoglycoside usage. J Antimicrob Chemother 1991;27(Suppl C):1-7.
26. Pradier C, Dunais B, Carsenti-Etesse H, Dellamonica P. Pneumococcal resistance patterns in Europe. Eur J Clin Microbiol Infect Dis 1997;16:644-7.
27. Nowak R. Hungary sees an improvement in penicillin resistance. Science 1994;264:364.
28. Kristinsson KC. Epidemiology of penicillin-resistant pneumococci. Nord Med 1996;111:103-8.
29. Gonzales R, Steiner JF, Sande MA. Antibiotic prescribing for adults with colds, upper respiratory tract infections, and bronchitis by ambulatory care physicians. JAMA 1997;278:901-4.
